# What Does the Talking?: Quorum Sensing Signalling Genes Discovered in a Bacteriophage Genome

**DOI:** 10.1371/journal.pone.0085131

**Published:** 2014-01-24

**Authors:** Katherine R. Hargreaves, Andrew M. Kropinski, Martha R. J. Clokie

**Affiliations:** 1 Department of Infection, Immunity and Inflammation, University of Leicester, Leicester, Leicestershire, United Kingdom; 2 Laboratory for Foodborne Zoonoses, Public Health Agency of Canada, West Guelph, Ontario, Canada; 3 Department of Molecular and Cellular Biology, University of Guelph, Guelph, Ontario, Canada; The Scripps Research Institute and Sorrento Therapeutics, Inc., United States of America

## Abstract

The transfer of novel genetic material into the genomes of bacterial viruses (phages) has been widely documented in several host-phage systems. Bacterial genes are incorporated into the phage genome and, if retained, subsequently evolve within them. The expression of these phage genes can subvert or bolster bacterial processes, including altering bacterial pathogenicity. The phage phiCDHM1 infects *Clostridium difficile*, a pathogenic bacterium that causes nosocomial infections and is associated with antibiotic treatment. Genome sequencing and annotation of phiCDHM1 shows that despite being closely related to other *C. difficile* myoviruses, it has several genes that have not been previously reported in any phage genomes. Notably, these include three homologs of bacterial genes from the accessory gene regulator (*agr*) quorum sensing (QS) system. These are; a pre-peptide (AgrD) of an autoinducing peptide (AIP), an enzyme which processes the pre-peptide (AgrB) and a histidine kinase (AgrC) that detects the AIP to activate a response regulator. Phylogenetic analysis of the phage and *C. difficile agr* genes revealed that there are three types of *agr* loci in this species. We propose that the phage genes belonging to a third type, *agr*3, and have been horizontally transferred from the host. AgrB and AgrC are transcribed during the infection of two different strains. In addition, the phage *agrC* appears not to be confined to the phiCDHM1 genome as it was detected in genetically distinct *C. difficile* strains. The discovery of QS gene homologs in a phage genome presents a novel way in which phages could influence their bacterial hosts, or neighbouring bacterial populations. This is the first time that these QS genes have been reported in a phage genome and their distribution both in *C. difficile* and phage genomes suggests that the *agr3* locus undergoes horizontal gene transfer within this species.

## Introduction

The incorporation of host DNA into phage genomes is reported to occur across diverse bacterial species, and such acquisition of bacterial genes facilitates phage evolution [1]. Although small, phage genomes have a high proportion of coding sequence relative to their size [2]. The extent by which viral genomes can increase is constrained physically by the dimensions of their virion particles in which their DNA is packaged, by fitness costs associated with phage production, and by their packaging strategy [3]. Although genetic material can be acquired via transduction and during DNA packaging, phage genomes are considered to be highly reduced and non-beneficial genes are lost through selective evolution [4]. Therefore, discoveries of bacterial gene homologs in addition to the “core” phage genome are interesting, as is the diverse nature of these host associated genes. These include the photosynthetic genes *psbA* and *psbD* found in cyanophages [5] and a gene encoding a tubulin-like protein found in a *Pseudomonas* phage [6]. These genes are expressed during infective cycles and are thought to enhance phage production. The expression of PsbA and PsbD are suggested to increase intracellular resources during phage replication and the tubulin organises viral DNA replication within the cell, in both examples the number of phage progeny released is potentially increased. Importantly, phages can be a source of novel genetic material to a newly infected host, especially when present as a prophage resulting in lysogen conversion. Examples of this include the lysogen converting phage infecting *Vibrio cholera*, CTXΦ [7] and the *Escherichia coli* STX phages [8], which encode toxin genes that increase their hosts' pathogenicity.

The facultative anaerobe *Clostridium difficile* is a major pathogen in healthcare settings, causing antibiotic associated diarrheal disease which can be fatal [9]. Novel strains continue to emerge in clinical settings [10], and potential reservoirs of the bacterium include asymptomatic humans, wild and domesticated animals, and the natural environment (e.g. [11]–[15]). The species is genetically diverse and different strains can produce up to three toxins, TcdA, TcdB and CDT, which are major virulence factors [16]. Others virulence determinants include colonisation factors such as adhesins and flagella [17] as well as the production of endospores that allow transmission and persistence outside the gut environment [18].

*C. difficile* pathogenicity can also be altered by the differential expression of their virulence genes, controlled via quorum sensing (QS) which is a form of bacterial communication [19]. Through quorum sensing, cells communicate to the surrounding population via the release and detection of signalling molecules which elicit a physiological response. The first *C. difficile* genome to be sequenced, strain CD630, has genes from both known bacterial QS systems, the *luxS* and the *agr* [17]. The *luxS* system have been experimentally verified [20], shown to upregulate the transcription of toxin genes *tcdA* and *tcdB* [21] and to be involved in biofilm production [22]. The *agr* system is also active, the *agr* locus, *agr2*, regulates the expression of TcdA and several genes involved in virulence and colonisation [23].

Despite the high proportion of lysogenic *C. difficile* strains described (e.g. [24], [25]), the contribution prophages make to *C. difficile* virulence is largely unexplored but the Pathogenicity Locus (PaLoc), encoding TcdA and TcdB, has been suggested to have a phage origin [26]. Several phages that are able to access a lytic lifecycle have been sequenced, but all encode integrases and show evidence of a temperate lifecycle [26]–[31]. Although none of these phages encode recognised toxins, some have been shown to influence host toxin production during infection but the mechanisms are unclear [30], [32].

To investigate how a phage from an environmental strain of *C. difficile* may contribute to host biology, we performed whole genome sequencing on the temperate phage phiCDHM1. Following the discovery that this phage has homologs of *agr* genes, their phylogeny was investigated with reference to homologs in sequenced *C. difficile* strains. To determine their stability during infections, their presence and transcription were probed for both lytic and lysogenic lifecycles. Lastly, a PCR based assay was used to establish if these phage encoded *agr* genes are widespread in our environmental strain collection.

## Methods

### Genome sequencing and annotation

PhiCDHM1 was isolated from strain CD105HS6 and propagated in a lytic manner on strain CD105HE1 to a high titre (10^10^ PFU). Genomic DNA (gDNA) was extracted using phenol∶chloroform and quantified on a Nanodrop 2000 (ThermoScientific, U.K.). The gDNA was sequenced using Roche 454 sequencing with a coverage of approximately 300×, reads were assembled using Phred/Phrap [33], [34] into two contigs. The genome was visualised in Artemis Genome Browser and Annotation Tool [35]. CDSs were identified using GeneMark.hmm 2.0 [36]. The contigs were joined by PCR using primers designed using Primer3v0.4.0 [37] as follows; 003AR 5′-TCACAAGCCTCAATTGCATTA-3′ and 004AR 5′-TGGCATTATTGTTAACAGCATCA-3′ which amplifies a 456 nt product and 003BF 5′-TTTGATATGAACAATGAAAATGAACA-3′ and 004BF 5′-TCCATATACTCATCGGAATTTTCA-3′ producing a 689 product. PCR reactions were performed in 50 µl, containing template DNA, 4 µM forward and reverse primers, 0.25 mM dNTPs, 3 mM MgCl_2_, 1× Biotaq buffer and 0.5 U of BioTaq DNA polymerase (Bioline, U.K.). Amplification conditions were: 95°C for 5 min, 30 cycles of 95°C for 30 sec, 48°C for 30 sec, 72°C for 60 sec, with a final extension of 5 min at 72°C.

Annotation was performed by searching the ORF aa sequences against the NCBI online nr/nt database using BLASTP, Pfam and Uniprot (04/2011). Protein domains were also identified using the NCBI Conserved Domain Database [38] and InterPro Scan (EBML accessed at http://www.ebi.ac.uk/Tools/InterProScan/ l). The genome was scanned for tRNAs using tRNAScan-SE 1.2 [39].

The NTPase and AgrC genes were fragmented into partial CDSs and were re-sequenced using Sanger sequencing. Primers to target the phage *agrC* were used: WHKF 5′-AGGATTTGTAATCCATAGGAACAT-3′ and WHKR 5′-TTTTCGfTTCGTTTTATTATTACAGTTT-3′ which have an expected 1657 bp product. Also, primers to target Orf85, a predicted NTPase gene, were used; NTPaseF 5′-CGCAAGTTACTGAAAAACTCCA-3′ and NTPaseR 5′-TTTCTCCCAATTTTTACACTGTTGA-3′ which amplify an 840 bp product. PCR reactions were carried out in 50 µl volumes containing DNA template, 4 µM forward and reverse primers, 0.25 mM dNTPs, 3 mM MgCl_2_, 1× PCR buffer and 0.5 U of BioTaq polymerase. Amplification conditions were: 95°C for 5 min, 30 cycles of 95°C for 30 sec, 48°C for 60 sec, 72°C for 120 sec, with a final extension of 5 min at 72°C. All products were visualised on a 1% agarose gel prepared in 1× TAE (Tris-acetate-EDTA pH 8) buffer stained with GelRed and run at 90 volts for 1 hr in TAE buffer alongside a 1 kbp molecular marker, GeneRuler (Fermentas, U.K.). Sanger sequencing was performed on gel-purified PCR products using the QIAquick Gel Extraction kit (Qiagen, U.K.) following manufacturer's instructions. Sequencing was carried out at GATC Biotech Ltd (U.K.). Data was analysed using Chromas v1.45 and Clustal Omega [40]. The linear genome map was generated using DNAplotter [41]. Statistical analysis was performed in Excel Microsoft Office 2007 using Single Factor ANOVA. Genome comparisons were performed using ACT [42] and EasyFig v2.1 [43].

### Phylogenetic analysis of phage *agr* genes

Genes homologous to *agrB* and *agrC* in sequenced *C. difficile* strains were identified using their translated sequences to search the NCBI nt/nr database with the BLASTP algorithm (Oct 2011). Homologs of *agrD* were identified by manually searching for candidate genes immediately upstream or downstream the *agrB* and *agrC* genes in deposited *C. difficile* genomes (Table S1). The amino acid sequences for each gene were aligned using the MUSCLE/Alignment Explorer in Molecular Evolutionary Genetics Analysis (MEGA) version 5.05 [44]. Maximum Likelihood (ML) phylogenetic analysis was performed, with parameters set for the Jones Taylor Thornton (JTT) nucleotide substitution model [45], with invariant rates, using all sites and Close-Neighbour-Interchange (CNI) for tree inference and bootstrapped with 500 replicates [46]. Alternative trees were also constructed using the Poisson nucleotide substitution mode, Neighbour Joining and Minimum Evolution phylogenetic analyses for comparison. ML phylogenetic analysis was also performed on sequences aligned using CLUSTALW/Alignment Explorer in MEGA v5.01. Trees topologies remained conserved, showing the same clustering of taxa, but branch lengths differed slightly between analyses.

### Transcription of the phage *agrB* and *agrC* genes in culture

Cultures of two *C. difficile* strains infected with phiCDHM1 were assayed to establish whether these genes are transcribed during the lytic and lysogenic lifecycle; the native lysogenic strain CD105HS6, a generated lysogenic strain CD105HE1, a lytic infection of CD105HE1 and an uninfected CD105HE1. Cultures from single colonies were grown in Brain Hearth Infusion broth (BHI: Oxoid, U.K.) and incubated at 37°C under anaerobic conditions (10% hydrogen, 10% carbon dioxide and 80% nitrogen gases) in a MiniMACS MG250 anaerobic chamber (Don Whitley Scientific, U.K.) overnight. Cultures were standardised using BHI to an OD550 nm of 1, and 1 ml used to inoculate 45 ml BHI. Once cultures reached an OD550 nm of 0.4, the CD105HE1 culture was diluted by a factor of 10 and phiCDHM1 added at an MOI of 10. Cultures were incubated for 30 min and then centrifuged at 3,400 *x*g for 10 min at 4°C. The pellet was snap frozen in liquid nitrogen and stored at 80°C until processing. After thawing the pellet on ice, RNA was extracted using the Maxwell 16 Total RNA kit (Promega, U.K.) in a Maxwell 16 machine following the manufacturer's guidelines. Additional DNase treatment was performed using Turbo DNase (Life Technologies, U.K.) according to the manufacturer's guidelines. DNA contamination was detected using PCR with primers that target the 16S rRNA gene as described by Rinttilä et al [47]. Purified RNA was quantified using an RNA Nano chip with the RNA 6000 Nano kit (Agilent Technologies, U.K.) on an Agilent 2100 Bioanalyzer (Agilent Technologies).

Synthesis of cDNA was performed using the RevertAid first strand cDNA synthesis kit (Fermentas, U.K.) with 1 µm of RNA and the random hexamer primers following the manufacturer's guidelines. The transcription of *agrB*, *agrC*, a predicted CI-like repressor (Orf76), predicted NTPase (Orf84) and predicted structural protein containing a baseplate J protein domain (Orf27) was determined using PCR. Primers were designed using Primer3v0.4.0 and oligonucleotide sequences provided in Table S2. AgrD was not included as its short length meant that suitable primers could not be designed. As a control, the primer set targeting the 16S rRNA gene was also used to check cDNA synthesis had occurred (data not shown). PCR reactions were performed separately for each primer set in 25 µl volumes with 1 µl of template cDNA, 0.6 mM of forward and reverse primer, 2 mM dNTPs, 1 volume of 10× BIOTAQ buffer, 0.5 U of BIOTAQ and 2 mM MgCl_2_. PCR conditions were 94°C at 5 min then 30 cycles of 94°C for 30 seconds and 55°C for 1 min. Products were separated using gel electrophoresis in TAE buffer after loading on to TAE 1% Helena Agarose gels with 6× DNA loading dye (Thermo Scientific, U.K.) and 10 µl of 1 kbp molecular marker (GeneRuler, U.K.) loaded for size comparison. Electrophoresis was conducted at 90 volts for 60 min and gels were visualised using SynGene software.

### Detection of phage specific *agrC* in environmental *C. difficile* genomes

Environmental *C. difficile* strains were routinely cultured in Fastidious Anaerobic Broth (FA: BioConnections, U.K.) or BHI under anaerobic conditions as above. DNA was extracted using Chelex 100 (Bio-Rad, U.K.) according to manufacturer's protocols. The two primer sets, 003AR/004AR and WHKF/WHKR, were used to screen *C. difficile* isolates to indicate presence and integration of *agrC* either in a phiCDHM1-like prophage. Primers 003AR/004AR are positioned inside *agrC* and are internal, whereas WHKF/WHKR are external, as are positioned in an upstream flanking sequence and in the *agrB* gene which is immediately downstream of *agrC* (Figure S2).

## Results

### Genome features of phiCDHM1 include bacterial homologs of the *agr* QS system

**Figure 1:**
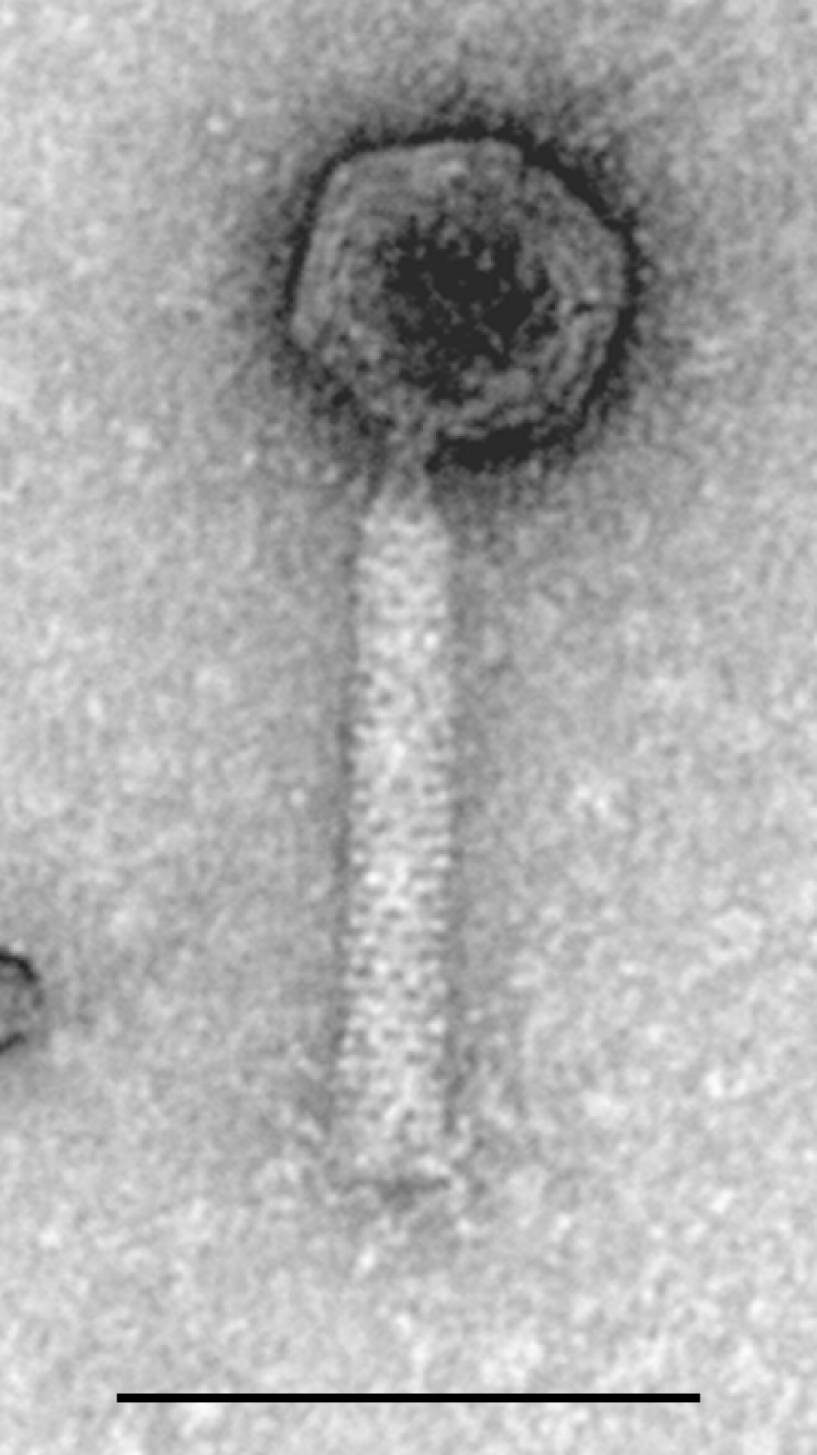
Particle morphology of phiCDHM1. TEM analysis of phiCDHM1 shows it to belong to the *Myoviridae* with an icosahedral capsid ∼60 nm, contractile tail sheath ∼110 nm length and ∼20 nm diameter and visible tail fibers. Scale bar is 100 nm.

**Figure 2:**
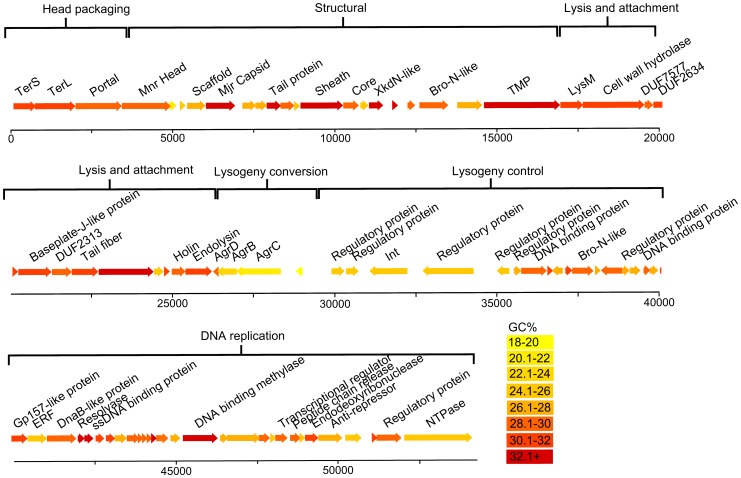
Genome map of phiCDHM1. Linear map showing position of CDSs to scale and annotated with predicted function. CDSs are coloured according to their GC content as shown in key.

The myovirus phiCDHM1 genome (Figure 1) was sequenced and found to be 54,279 bp with an average GC content of 28.4%. The annotation of the genome has been oriented to start with the small subunit terminase as Orf1, in order to be consistent with the first annotated *C. difficile* phage, ΦCD119 [27]. 84 putative CDSs were identified, 75 of which are on the sense strand, and the predicted coding sequence accounts for 88.4% of the genome. A linear plot of phiCDHM1 is shown in Figure 2, with CDSs coloured according to their average GC%. No tRNAs were identified using tRNAScann-SE 1.2.

The genome is highly mosaic but shares a homologous modular arrangement by gene function in common with the *C. difficile* myoviruses φC2 and ΦCD119, which also share a similar particle morphology and genome size [26], [27]. Relatedness to known phages can be inferred by genes which are conserved between other *C. difficile* phage and prophage genomes. The presence of a DNA replication cassette that is characteristic of the phiCD119-like *C. difficile* myoviruses, places phiCDHM1 within this taxonomic group [48].

In addition to the conserved phage genes, we identified four homologs of bacterial genes that are not present in the other phage genomes. These encode a predicted NTPase and three proteins involved in the *agr* QS pathway. The NTPase gene is located at the 3′ end of the DNA replication region and the QS genes are immediately after the lysis genes, on the anti-sense strand upstream of the integrase gene.

The predicted NTPase (Orf84) contains a NACHT domain (PFam CL0023, E value 2.2e-05) and has homology to hypothetical proteins encoded in *Clostridium kluyveri* strains. Although other phages encode proteins with predicted NTPase function, for example G166_gp42 in *Clostridium sporogenes* phage Φ8074-B1, we could find no entry of a phage gene with an annotated NACHT domain in NCBI although a DELTA blast search identified homologous sequences in the viral NCBI db.

The predicted QS genes share sequence homology and shared protein domain motifs with bacterial genes of the *agr* QS system encoded in *C. difficile* strains, and are predicted to be *agrD*, *agrB* and *agrC*. The protein encoded by gene *agrD* contains the characteristic P-X-X-P motif (where AgrB binds [49]) which is located between aa residues 35 and 38. The *agrB* gene product has an AgrB domain (PF04647) and the *agrC* gene encodes a protein with a HATPase_C domain (PF02518). This protein domain is a GHKL (gyrase, Hsp90, Histidine Kinase, MutL) domain which is characteristic of histidine kinases, including AgrC but the phage AgrC does not contain an identified receptor domain. It has been annotated as *agrC* due to its proximity to the *agrB* and *agrD* homologs. To our knowledge, this is the first time that these three *agr* genes have been reported in a phage genome.

The *agr* system is found throughout Gram-positive bacterial species although the content and organisation of loci vary. The first *agr* locus to be described was in *Staphylococcus aureus* and it encodes AgrD, AgrB, AgrA and AgrC [49]. The gene *agrD* encodes a pre-peptide that is cleaved post-translationally into an autoinducing peptide (AIP). The cleavage of AgrD is performed by AgrB and the resulting AIP is released from the cell. Exogenous AIP is recognised by the membrane bound AgrC, and a response is elicited following phosphorylation of the response regulator AgrA by AgrC. Importantly, no associated response regulator was identified in the phage genome.

The phage *agr* genes have a significantly lower average GC content (24.37%) than the genes in the structural (31.4%), lysis and attachment (30.16%) and DNA replication (30.2%) modules (p values of 0.0093, 0.011 and 0.026 respectively) and lower than the average of all genes in the phiCDHM1 genome (28.7%, p value of 0.019). Furthermore, the GC content of *agrC*, *agrB* and *agrD* homologs in strain NAP08 (accession GCA_000164175.1) are comparative to the phage genes; 20.4% to 21.1% in *agrC*, 21.8% to 22.2% in *agrB* and 28.1% to 25.9% in *agrD*, respectively. Although the total GC content of strain NAP08 is higher than these genes, at 28.9%, it is known that the GC% of strain CD630 varies throughout the genome and the average is elevated due to the presence of multiple mobile genetic elements [17]. It therefore seems likely that the phage QS genes have a host origin which would explain their lower average GC%.

### Phylogenetic analyses of the phage *agr* genes reveal evolutionary divergence and horizontal transfer in *C. difficile* strains

**Figure 3:**
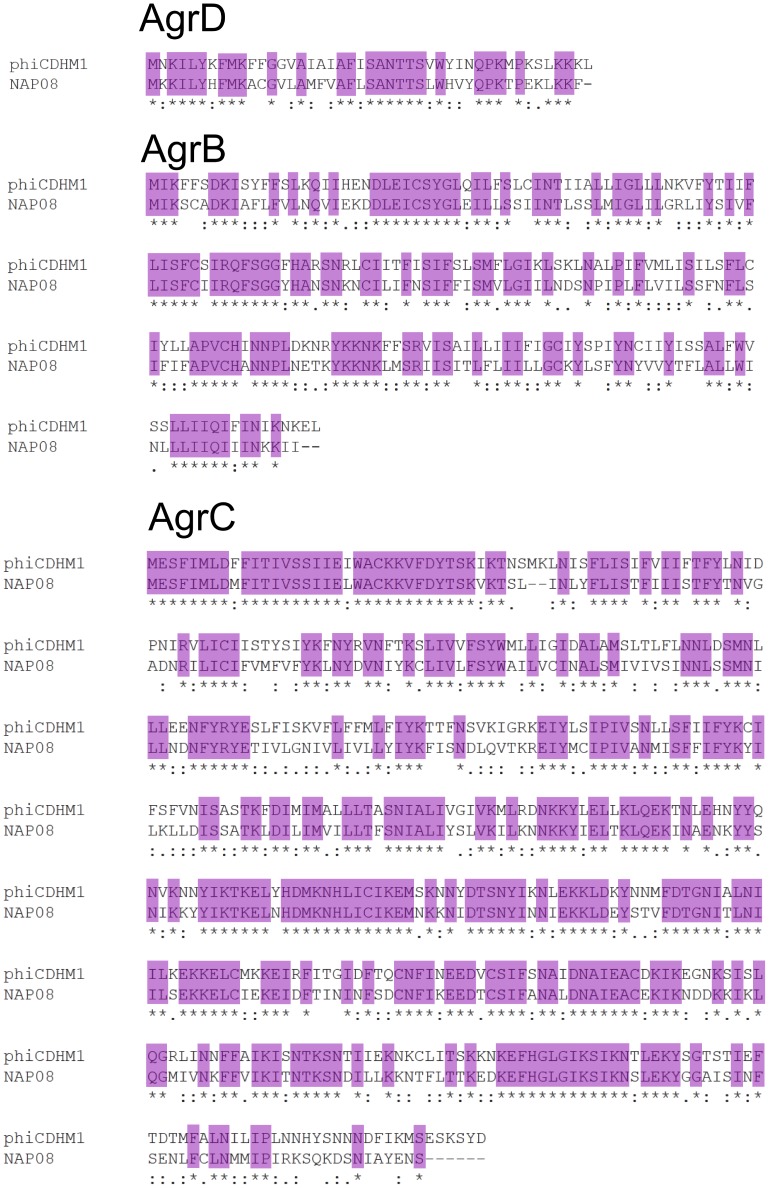
Alignments of the *agrD*, *agrB* and *agrC* genes of phiCDHM1 and *C. difficile* strain NAP07. Alignments between phiCDHM1 (top sequence in all) and *C. difficile* strain NAP07 (bottom sequence in all) at the aa level. Purple shading highlights identical residues. Top: the *agrD* genes, 45 and 46 aa long respectively, share a 59% identity. Middle: the *agrB* genes, 197 and 195 aa long respectively, share 54.8% identity. Bottom: the *agrC* genes, 453 and 445 aa long respectively, share 61% identity.

**Figure 4:**
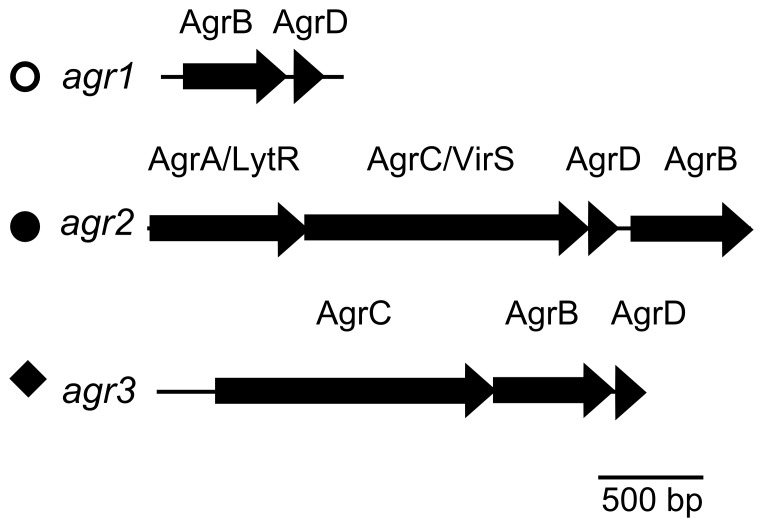
Three types of *agr* loci are present in different strains of *C. difficile* and phiCDHM1. Three types of *agr* loci have been identified. The *agr1* locus encodes AgrB and AgrD: the a*gr2* locus encodes AgrA (containing a LytTR protein domain), AgrC (with homology to VirS), AgrD and AgrB: the *agr3* locus encodes the AgrC, AgrB and AgrD. Symbols; empty circle, full circle and diamond are in reference to the taxa clusters in the ML phylogenetic analysis in Figures 5–7.

To investigate the origins of these QS genes in a phage genome their sequence similarities and phylogenetic relationships to their closest bacterial homologs were determined. The aa sequence similarities between the phage *agrD*, *agrB* and *agrC* and homologs in strain NAP07 are 56%, 58% and 61%, respectively (Figure 3). Results of the BLASTP searches showed that *C. difficile* strains in the NCBI database encode different types of *agr* loci. Multiple *agr* gene carriage was previously reported for R027 strains which encode two loci, *agr1* and *agr2* [50] whereas strain CD630 only encodes *agr1* [17]. The gene content differs between loci: *agr1* has *agrD* and *agrB* and *agr2* encodes homologs of AgrA, AgrC, AgrD and AgrB [17], [50]. We report the presence of a third locus, *agr3*, in phiCDHM1 which has *agrC*, *agrB* and *agrD* (Figure 4). The *agr3* locus is also present in *C. difficile* strains NAP07, NAP08 and QCD-23m63, all of which also encode *agr1*.

**Figure 5:**
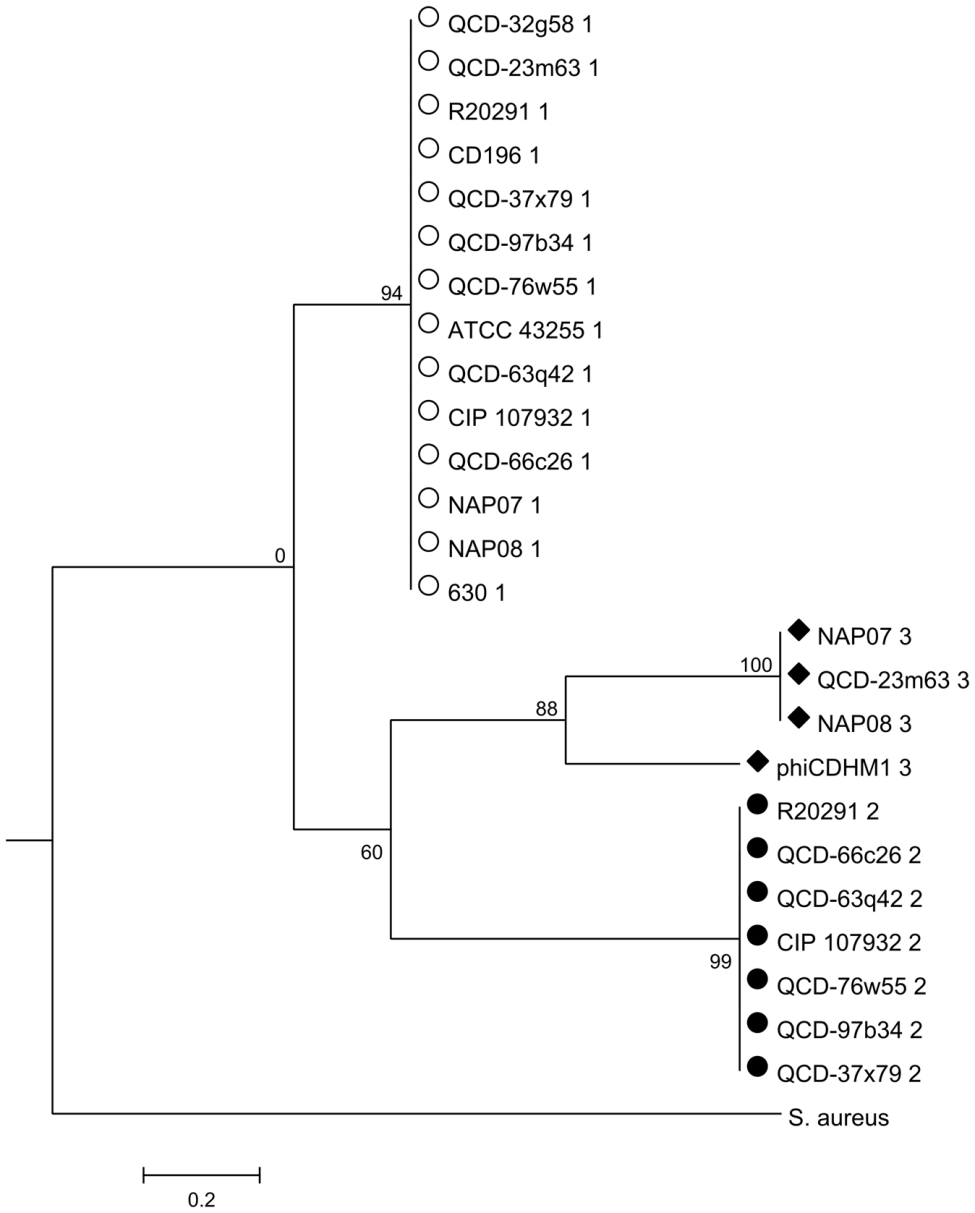
ML phylogenetic analysis of the *agrD* gene from phiCDHM1 and *C. difficile* strains. Phylogenetic analysis was performed on the *agrD* genes of phiCDHM1 and sequenced *C. difficile* strains in the NCBI genome db (Oct 2011) and *agrD* of *S. aureus subsp. aureus MRSA252*. The translated sequences were aligned with MUSCLE and ML analysis performed using parameters set for the JTT nucleotide substitution model, with invariant rates, using all sites and CNI for Tree Inference and bootstrapped with 500 replicates in MEGAv5.01. Symbols correspond to those shown in Figure 4 and indicate the type of *agr* locus in which the gene is present (either *agr1*, *agr2* or *agr3*). Taxa are abbreviated to strain names and number indicates locus type.

**Figure 6:**
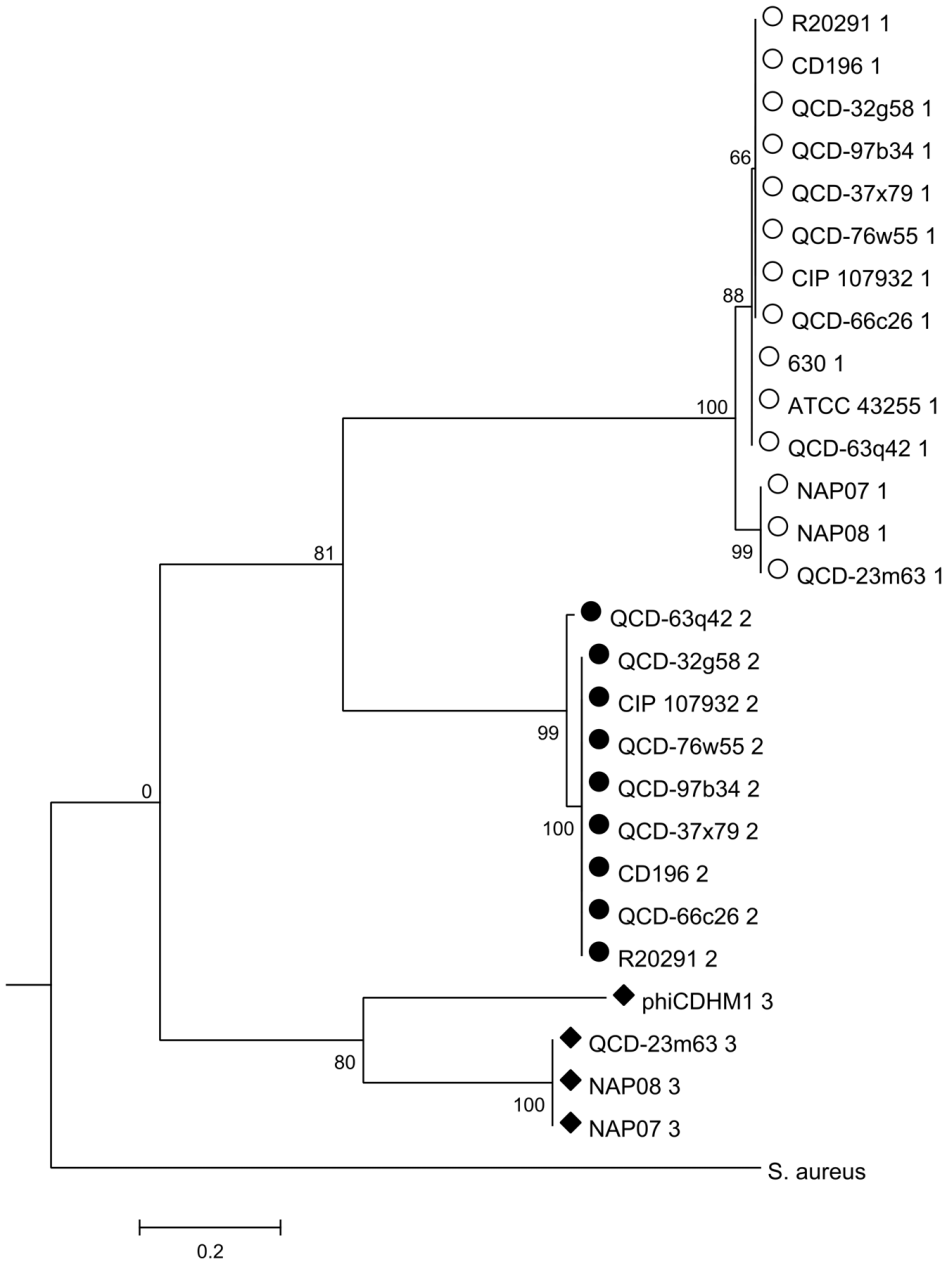
ML phylogenetic analysis of *agrB* genes from phiCDHM1 and *C. difficile* strains. Phylogenetic analysis was performed on the *agrB* genes of phiCDHM1, sequenced *C. difficile* strains in the NCBI genome db (Oct 2011) and *agrB* of *S. aureus subsp. aureus MRSA252*. The translated sequences were aligned with MUSCLE and ML analysis performed using parameters set for the JTT nucleotide substitution model, with invariant rates, using all sites and CNI for Tree Inference and bootstrapped with 500 replicates in MEGAv5.01. Symbols correspond to those shown in Figure 4 and indicate the type of *agr* locus in which the gene is present (either *agr1*, *agr2* or *agr3*). Taxa are abbreviated to strain names and number indicates locus type.

**Figure 7:**
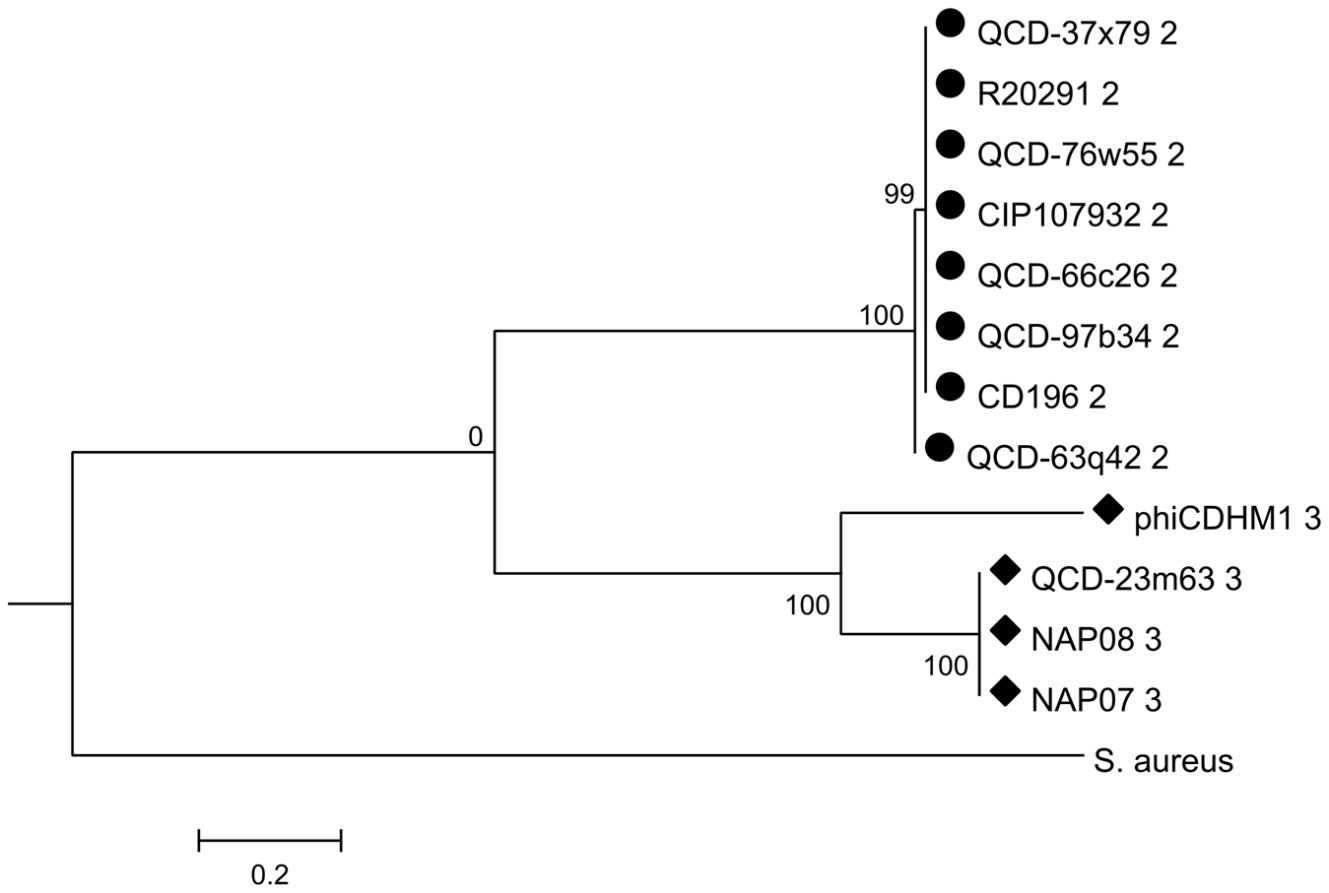
ML phylogenetic analysis of related *agrC* genes from phiCDHM1 and *C. difficile* strains. Phylogenetic analysis was performed on homologs of *agrC* in the *agr1* and *agr3* loci of *C. difficile* strains in the NCBI genome db (Oct 2011) and the *agrC* gene from *S. aureus subsp. aureus MRSA252*. The translated sequences were aligned with MUSCLE and ML analysis performed using parameters set for the JTT nucleotide substitution model, with invariant rates, using all sites and CNI for Tree Inference and bootstrapped with 500 replicates in MEGAv5.01. Symbols correspond to those shown in Figure 4 and indicate the type of *agr* locus in which the gene is present (either *agr1*, *agr2* or *agr3*). Taxa are abbreviated to strain names and number indicates locus type.

The phylogenies for each gene were investigated and the resulting trees correspond to the *agr* loci types: *agrD* (Figure 5); *agrB* (Figure 6) and *agrC* (Figure 7). The *agrB* and *agrD* genes cluster into three groups which correspond to the *agr* types, *agr1*, *agr2* and *agr3*. The tree for *agrC* has fewer taxa, because this gene is not present in *agr1*, and it shows that the genes in *agr1* and *agr3* cluster into two distinct clades. Branch lengths are similar between the *agrD* and *agrB* trees and the bootstrap values for each *agr* loci cluster are all above 80. However, the relative relationships of the loci are not resolved. For *agrB*, the clusters corresponding to the genes in *agr1* and *agr2* may be more related to each other than to those of *agr3*, as supported by a bootstrap value of 81, but in the analysis of *agrD* there is no inference of inter-locus relationship.

Importantly, despite the phage sequences clustering with those of the other *agr3* genes carried by *C. difficile* strains NAP07, NAP08 and QCD-23m63; they are genetically distinct as can be seen from the branch length distances, bootstrap values and the Clustal Omega alignments. Whether the genes in the phage *agr3* locus are therefore functionally distinct, or whether the AIP sequences are similar enough to be recognised by the bacterial AgrC of the *agr3* is unknown.

### Transcription of the phage encoded *agrB* and *agrC* during infection

PhiCDHM1 can infect strain CD105HE1 in a lytic and lysogenic manner. The transcription of the *agrB* and *agrC* genes was determined for both in addition to the native lysogen, strain CD105HS6. An uninfected culture of CD105HE1 was used as a negative control as it does not encode the phage *agr* genes. As expected, transcription of the bacterial 16S rRNA gene was detected in all four cultures (Figure S1). Transcription of the predicted structural gene Orf23 and the predicted repressor protein Orf76 were detected for all three phiCDHM1 infected cultures. These three cultures also showed transcription of the predicted NTPase (Orf84) and the phage *agrB* and *agrC* genes. Although not quantitative, there appears to be differential transcription between the cultures based on the relative abundance of product on the gels. This difference may be due to the level of lytic or lysogenic life cycle replication occurring in each culture, as phiCDHM1 can lysogenize strain CD105HE1, and can also be released spontaneously from CD105HS6. Further work in this laboratory is currently being conducted to establish the transcription dynamics in these cultures in a quantitative manner.

### Detection of the phage *agrC* gene in environmental *C. difficile* isolates

Two primer sets were used to detect the carriage of phage-specific *agrC* in isolates of *C. difficile* (Table 1 and Figure S2). The internal primer set determines the presence of *agrC*, and the external set was designed to test whether it is present in phiCDHM1-like prophages as the forward primer begins −174 nucleotides upstream of *agrC* and is specific to phiCDHM1. To design these primers, the flanking sequences of the *agr* locus were examined, and 300 nts upstream of *agrC* shares 85% nt similarity between phiCDHM1 and NAP08, but the flanking sequence 50 nts downstream (avoiding overlap with the endolysin gene) of *agrD* is not homologous. Due to the large size of the cassette, the reverse primer is located in *agrB*. Three isolates were positive for the expected sized product following amplification with the internal primer set; CD105HS27 (R078) and CD105HS31 (R046) and, as expected, CD105HS6 which was used as a positive control. Only isolate CD105HS6 had the expected product amplified by the external primer set, suggesting that the gene has a different genetic background and may be present either on an unknown prophage or on the bacterial chromosome of CD105HS27 and CD105HS31. It does however show that this gene is not confined to this one phage genome.

**Table 1:** Isolate screen for phage *agrC* using internal and external primer sets.

Isolate	Ribotype	1	2
CD105HS14	010	−	−
CD105HS15	010	−	−
CD105HS16	010	−	−
CD105HS9	010	−	−
CD105HS23	001	−	−
CD105HS24	001	−	−
CD105HS25	001	−	−
CD105HS12	001	−	−
CD105HS22	220	−	−
CD105HS2	220	−	−
CD105HS6	220	+	+
CD105HS17	002	−	−
CD105HS7	002	−	−
CD105HS18	031	−	−
CD105HS19	031	−	−
CD105HS20	005	−	−
CD105HS10	005	−	−
CD105HS26	078	−	−
CD105HS27	078	+	−
CD105HS3	046	−	−
CD105HS31	046	+	−
CD105HS4	014	−	−
CD105HS5	021	−	−
CD105HS8	027	−	−
CD105HS1	012	−	−
CD105HS21	106	−	−
CD105HS11	Unknown	−	−
CD105HS28	Unknown	−	−

1 = internal primer set.

2 = external primer set.

This phage can access a range of *C. difficile* hosts as demonstrated by turbid lysis (indicating lysogenic infection) which was observed for 12.9% of 160 isolates tested and include isolates belonging to six ribotypes. Furthermore, ten generated lysogens of the strain CD105HE1 were tested with the internal primer set; 003AR/004AR and all produced a PCR product, indicating that this region is typically retained following lysogeny.

## Discussion

### PhiCDHM1 belongs to the phiCD119-like group of *C. difficile* myoviruses, but key genetic differences include the presence of quorum sensing genes in its genome

The genome of phiCDHM1 is closely related to those of the *C. difficile* myoviruses phiC2 and phiCD119 [26]. The phage has putative genes that are involved in essential functions in the phage temperate lifecycle, such as head packaging, morphogenesis, attachment, lysis, lysogeny control and DNA replication. While the genome follows a similar overall architecture in functional modules and many genes are conserved between these phages, it shows evidence of extensive mosaicism based on individual gene similarities. This has been frequently observed in phages infecting other species, for example throughout the mycobacteriophages [51]. Surprisingly though, phiCDHM1 encodes predicted homologs of AgrD, AgrB and AgrC. Whilst the scenario of phages acquiring genes from their bacterial host genome is well documented (for example [5], [52]), these genes are the first example of a QS cassette to be discovered in a phage genome.

### Diversity and evolutionary origin of phage *agrD*, *agrB* and *agrC* genes

Phylogenetic analysis of each gene at the aa level found that they cluster together with other bacterial genes from the same type of *agr* locus and we suggest the phage *agr* genes have a host origin and evolved within the phage genome or represent a subtype. Interestingly, all of the *C. difficile* strains included in our analysis have the *agr1* locus, but some have an additional locus, either *agr2* or *agr3*, which indicate that the different loci have accessory functions within *C. difficile*.

The transfer of these genes throughout the *C. difficile* population could involve horizontal gene transfer (HGT), as well as phage infection. The *agr3* genes in *C. difficile* strains NAP07 and NAP08 are not located in prophages, but predicted transposases and a phage-like integrase gene are in close proximity and this could be a mobile *agr* locus. Our findings are consistent with those of another study, which mapped AgrB sequences to a 16S rRNA tree for 384 species of *Firmicutes* [53]. In general, AgrB showed a vertical pattern of evolution, except in *Clostridium acetobutylicum* which was most related to that of *Listeriaceae* and led researchers to conclude evolution of the gene via HGT may have occurred. We found that the phage specific *agrC* gene is present in genetically diverse isolates and appears to be on a phage distinct from phiCDHM1 or, alternatively, on the bacterial chromosome in these isolates. Our data shows that, although not widespread, the exchange of the *agr* genes in Clostridia via HGT occurs more commonly than previously thought.

### Carriage of *agr* genes in a phage genome presents a novel mechanism for phages to influence their bacterial hosts

The phage *agr* genes group closely with their bacterial homologs, but are distinct (Figure 3). They may have evolved within the phage genome, or represent a previously undiscovered subtype of the *agr3* locus. The genes are retained during lytic and lysogenic replication and are transcribed so are likely to have a functional role. While this is the first time that the *agr* QS cassette has been identified in a phage genome, there are examples where phages and QS systems interact.

In one study, native soil bacterial populations were shown to release phages when they were exposed to several species variants of the signalling molecule, N-acyl homoserine lactone, from the *luxS* QS system [54]. Whether phages can actively ‘listen in’ to this signal is unknown, but there are several sequenced phages in the NCBI database that encode gene homologs of LuxR, the response regulator, as they contain either LuxR_C_like or HTH_LUXR protein domains. These are characteristic of transcriptional regulators, including LuxR and they are found both in known temperate and plasmid-like phages (e.g. [55]–[57]) as well as in virulent phages [58].

In contrast to listening in, one phage, φPLPE which infects *Iodobacter*, may instead block out the *luxS* QS signal, as it encodes a putative acylhydrolase, which in the bacterial homolog degrades the N-acyl homoserine lactone signal molecules [59]. An example of why a phage may want to block the signal of the QS system is seen in *Escherichia coli* and lambda interactions. The phage receptor molecules for lambda are down-regulated via the *luxS* system and so inhibiting this would presumably allow a successful infection for the phage [60].

There are fewer examples of linking phages and the *agr* QS system, but interestingly three phage genomes contain genes with a LytTR protein domain (and so may be homologous to the response regulator, AgrA) and may therefore have the capacity to ‘listen in’ to this system. They are all phages that infect *Pseudomonas spp*, phage Lu11 [61], phage vB_PaeS_PMG1 (NC_016765.1) and phage D3 [62]; two of which encode predicted integrases.

Clearly phages could benefit from interacting with their bacterial QS systems through listening in and blocking the signals and the phage phiCDHM1 is the first example of a phage with the genes necessary to do the ‘talking’ instead. Further analysis of these three genes has identified highly similar predicted CDSs (98–100% identity) in several *C. difficile* strains in a WGS project recently deposited in NCBI and include those isolated from asymptomatic, acute and relapse patients (Table S3). Where possible to distinguish, it can be seen that these genes are in prophage-like sequences, and in one strain the entire prophage has been assembled on one contig, strain DA00261. An ACT comparison of this prophage sequence to phiCDHM1 shows they are homologous but not identical (data not shown). By performing a DELTA Blast of *agrB* against the viral db at NCBI (Oct 2013) we also found a putative cassette of *agr* genes in three *Paenibacillus* phage genomes; phage Davies, phage Emery and phage Abouo (accessions KC595518, KC595516 and KC595517), each with a predicted AgrB, putative AgrD and one or two predicted membrane proteins which may be homologs of AgrC although lack a HTPase_C domain. The genes have low aa sequence similarity to the phiCDHM1 homologs, and are also distinct from one another. The predicted AgrB homologs are 28.5%, 27.9% and 25.9% similar to phiCDHM1 respectively; the putative AgrC homologs are 18.5%, 20.4% and 19.1% respectively and the AgrD homologs are 23.7%, 26.3% and 27.5% respectively, following alignment in Clustal Omega. The orientation of these genes are conserved between the *Paenibacillus* phages, but differ from phiCDHM1, and their genomes are similarly divergent (Figure S3). However, like phiCDHM1, these phages all encode integrases suggesting they can access the lysogenic lifecycle. The observation of these genes in other phage genomes shows this this phenomenon is not confined to *C. difficile* and supports our hypothesis that these genes are of functional importance.

Maintaining additional genes is resource costly, but as these genes are retained and transcribed it is likely that they are beneficial so why this phage encodes such a large and resource expensive cassette is of interest. As no response regulator gene was identified in the phage genome, we suggest that the phage signal is released, detected by its associated kinase and the signal relayed onto elicit a host mediated response, perhaps using AgrA in *agr2*. Three scenarios as to when a (pro)phage may evoke a QS coordinated response include but are not limited to 1) in playing a role in niche construction so using the QS genes as a weapon in intermicrobial wars, 2) as a population density-dependent lysogen conversion factor enhancing its host's fitness or 3) protection against secondary phage infection by, for example, altering a surface receptor. All three strategies would promote phiCDHM1 and its' host's survival and replication.

In the first scenario relating to niche construction, the phage encoded signal peptide could be released as an antagonist to reduce microbial competition for resources by causing lysis of neighbouring cells via phage induction. Depending on whether the signal is working on its own induction or unrelated phage induction, the phage may be co-ordinating its own release, or clearing unrelated lysogens which then become a food source for the phage host. The induced phages would also then be free to propagate and infect new hosts, also known as “kill the relatives” or lysogen alleopathy [63]–[65].

Secondly, the phage may be eliciting a response in its own host to promote fitness such as toxin or spore production. In *C. difficile*, the *agr2* system has been found to regulate fitness, including increasing toxin A production, using AgrA mutants [23]. Whether the *agr1* or *agr3* loci, which lack an AgrA, have similar roles is not known. Interestingly, *C. botulinum* also encodes two different *agr* loci and each evokes a different response; *agr*-1 modulates sporulation and *agr*-2 toxin production [66]. The phage *agr* loci could therefore have a different response than the *agr* loci of the host bacteria.

Lastly, it may serve as a defence mechanism. The signal could down regulate cell surface molecules to inhibit secondary phage infection. As mentioned previously, the *luxS* QS has been found to prevent phage infection in *E. coli because* as the signal decreases the number of phage lambda receptors on its cell surface protein [60]. Phages are known to encode genes that are predicted to be involved in secondary phage infection such as the *Clostridium* phage phiC2 which encodes an AbiF protein [26]. Using the QS system to prevent phage infection would be a new mechanism for phages to engage in phage resistance.

Whilst the action and consequences of these phage QS genes is unclear, their presence and transcription during infection in a lysogenic and lytic background presents an exciting method by which phages can manipulate their hosts. Work to investigate further these intriguing phage QS genes is ongoing in our laboratory.

## Data Access

The accession number for phiCDHM1 is HG531805.

## Supporting Information

Figure S1 **Transcription of phage genes including QS genes during phiCDHM1 infection.** PCRs were performed on cDNA using primers to target *agrB*, *agrC*, Orf76 (predicted repressor CI-like gene), Orf23 (the basteplate-J structural gene) and Orf84 (NTPase gene). cDNA was generated from phage phiCDHM1 infected culture of strain CD105HE1 and two lysogenic strains; CD105HS6 and a generated CD105HE1 lysogen, in addition to controls of uninfected CD105HE1 culture, genomic DNA from CD105HS6, CD105HE1 and phiCDHM1. Top L-R: PCR for *agrB*, lanes 1–9: GeneRuler 1 kbp, CD105HS6, CD105HE1, CD105HE1 lysogen, CD105HE1+phiCDHM1, CD105HS6 gDNA, CD105HE1 gDNA, phiCDHM1 gDNA, -ve control; PCR for *agrC* lanes, 1–9: GeneRuler 1 kbp, CD105HS6, CD105HE1, CD105HE1 lysogen, CD105HE1+phiCDHM1, CD105HS6 gDNA, CD105HE1 gDNA, phiCDHM1 gDNA, -ve control; PCR for Orf76 lanes, 1–9: phiCDHM1 gDNA, GeneRuler 1 kbp, CD105HS6, CD105HE1, CD105HE1 lysogen, CD105HE1+phiCDHM1, CD105HS6 gDNA, CD105HE1 gDNA, -ve control. Below L-R: PCR for Orf23, lanes 1–9: GeneRuler 1 kbp, CD105HS6, CD105HE1, CD105HE1 lysogen, CD105HE1+phiCDHM1, CD105HS6 gDNA, CD105HE1 gDNA, phiCDHM1 gDNA, -ve control; PCR for Orf84, lanes 1–9: GeneRuler 1 kbp, CD105HS6, CD105HE1, CD105HE1 lysogen, CD105HE1+phiCDHM1, CD105HS6 gDNA, CD105HE1 gDNA, phiCDHM1 gDNA, -ve control.
(TIF)

Figure S2 **Positions of the internal and external Primers to probe the carriage of phiCDHM1 specific** ***agrC*** **in** ***C. difficile*** **isolates.** Primer sets used in screening for the *agrC* with internal set 003AR/004AR (positions 27217–27237 and 27651–27673 bp) and external set WHKF/WHKR located at 004AR (positions 26808–26831 and 28439–28465 bp). WHKR is located in a non-coding region of the genome.
(TIF)

Figure S3 **Whole genome comparisons of phages with** ***agr*** **homologs.** The genome sequences of phages Emery, Davies, Abouo and phiCDHM1 are shown with corresponding tblastx comparisons between the genomes, performed in EasyFig v2.1. The locations of the putative *agr* homologs in their genomes are highlighted in pink. Scale is 1 kbp and blast similarity ranges shown in the key.
(TIF)

Table S1 Strains and accession numbers for *agr* genes used in phylogenetic analysis.
(DOCX)

Table S2 Primers used for phage gene transcription PCR assays.
(DOCX)

Table S3 Strains and accessions of *C. difficile* isolates with phage *agr* genes.
(DOCX)
