# Supplementary figures and images for: What Does the Talking?: Quorum Sensing Signalling Genes Discovered in a Bacteriophage Genome
Source: PLoS One. 2014 Jan 24;9(1):e85131. doi: 10.1371/journal.pone.0085131 (PMC3901668; doi:10.1371/journal.pone.0085131)

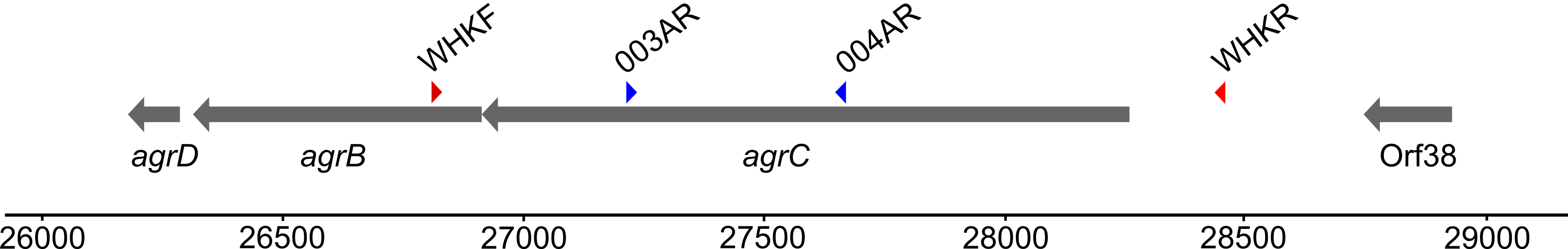

Supplement: Figure S2 — Positions of the internal and external Primers to probe the carriage of phiCDHM1 specific agrC in C. difficile isolates. Primer sets used in screening for the agrC with internal set 003AR/004AR (positions 27217–27237 and 27651–27673 bp) and external set WHKF/WHKR located at 004AR (positions 26808–26831 and 28439–28465 bp). WHKR is located in a non-coding region of the genome. (TIF) [file pone.0085131.s002.tif]

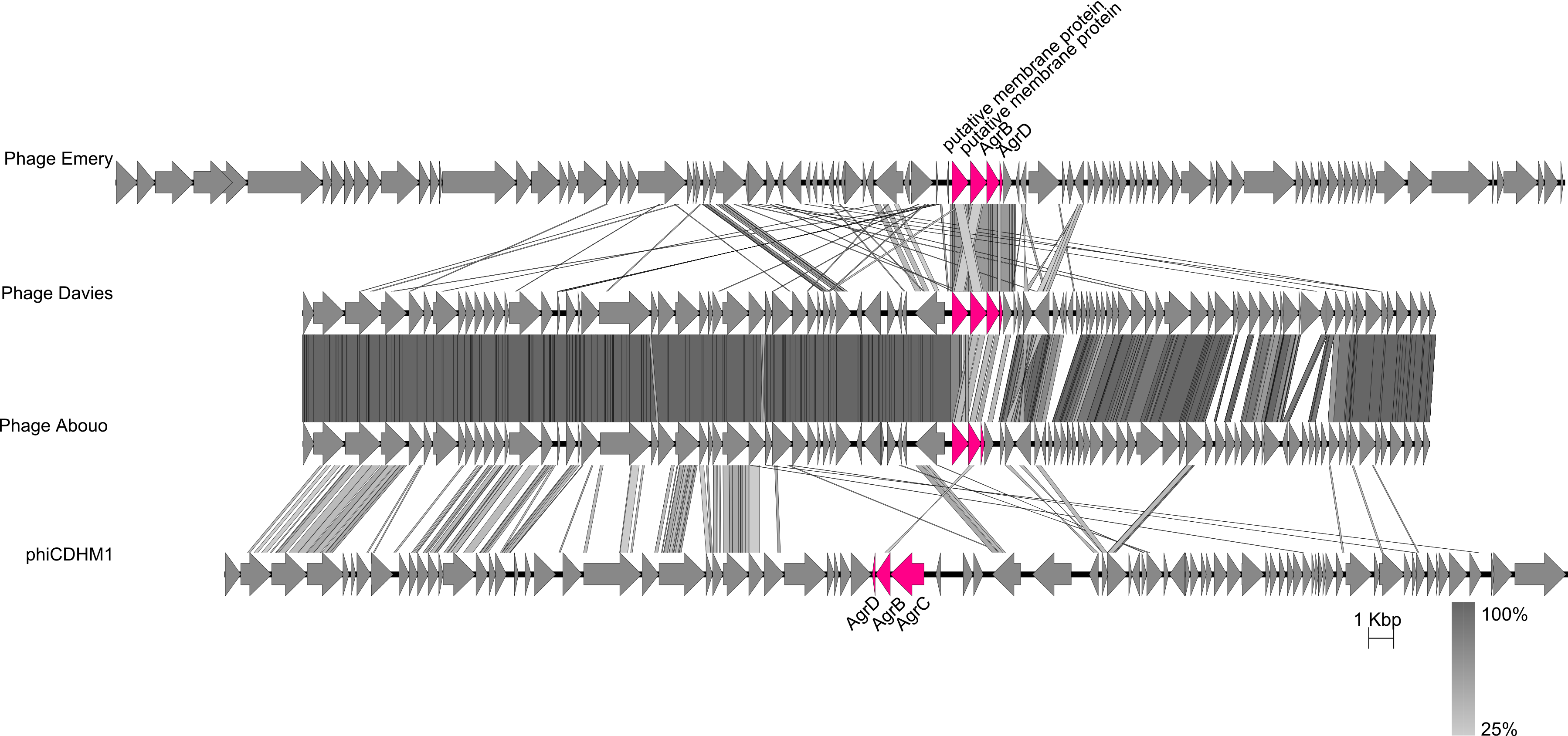

Supplement: Figure S3 — Whole genome comparisons of phages with agr homologs. The genome sequences of phages Emery, Davies, Abouo and phiCDHM1 are shown with corresponding tblastx comparisons between the genomes, performed in EasyFig v2.1. The locations of the putative agr homologs in their genomes are highlighted in pink. Scale is 1 kbp and blast similarity ranges shown in the key. (TIF) [file pone.0085131.s003.tif]
