# Supplementary material for: What Does the Talking?: Quorum Sensing Signalling Genes Discovered in a Bacteriophage Genome
Source: PLoS One. 2014 Jan 24;9(1):e85131. doi: 10.1371/journal.pone.0085131 (PMC3901668; doi:10.1371/journal.pone.0085131)
Supplement: Table S1 — Strains and accession numbers for agr genes used in phylogenetic analysis. (DOCX) [file pone.0085131.s004.docx]

| **Strain (*C. difficile*)** | **Type** | | **AgrB** |  | **AgrD** | | **AgrC/VirS** | | **LytTR** |
| --- | --- | --- | --- | --- | --- | --- | --- | --- | --- |
| QCD-66c26 | 1 | ZP_05273396.1 | | | ZP_05273397 | | ZP_05273398.1 | | ZP_05273399.1 |
| CIP 107932 | 1 | ZP_05323787.1 | | | ZP_05323788 | | ZP_05323789.1 | | ZP_05323790.1 |
| QCD-76w55 | 1 | ZP_05357647.1 | | | ZP_05357648 | | ZP_05357649.1 | | ZP_05357650.1 |
| QCD-97b34 | 1 | ZP_05386399.1 | | | ZP_05386400 | | ZP_05386401.1 | | ZP_05386402.1 |
| QCD-37x79 | 1 | ZP_05398745.1 | | | ZP_05398746 | | ZP_05398747.1 | | ZP_05398748.1 |
| CD196 | 1 | YP_003216155.1 | | | YP_003216156 | | YP_003216157.1 | | YP_003216158.1 |
| R20291 | 1 | YP_003219662.1 | | | YP_003219663 | | YP_003219664.1 | | YP_003219665.1 |
| QCD-63q42 | 1 | ZP_05331470.1 | | | ZP_05331471 | | ZP_05331472.1 | | ZP_05331473.1 |
| QCD-32g58 | NA | ZP_07407976.1 | | | ZP_07407977 | | ZP_07405550.1 | | |
| 630 | 2 | YP_001089263.1 | | | YP_001089262 | | |  | |
| QCD-63q42 | 2 | ZP_05330899.1 | | | ZP_05330898 | | |  | |
| ATCC 43255 | 2 | ZP_05351962.1 | | | ZP_05351961 | | |  | |
| QCD-66c26 | 2 | ZP_05272818.1 | | | ZP_05272817 | | |  | |
| CIP 107932 | 2 | ZP_05323209.1 | | | ZP_05323208 | | |  | |
| QCD-76w55 | 2 | ZP_05357066.1 | | | ZP_05357065 | | |  | |
| QCD-97b34 | 2 | ZP_05385822.1 | | | ZP_05385821 | | |  | |
| QCD-37x79 | 2 | ZP_05398164.1 | | | ZP_05398163 | | |  | |
| CD196 | 2 | YP_003215611.1 | | | YP_003215610 | | |  | |
| R20291 | 2 | YP_003219119.1 | | | YP_003219118 | | |  | |
| QCD-32g58 | NA | ZP_07407451.1 | | | ZP_07407450 | | |  | |
| QCD-23m63 | 2 | ZP_05402145.1 | | | ZP_05402144 | | |  | |
| NAP08 | 2 | ZP_06894136.1 | | | ZP_06894137 | | |  | |
| NAP07 | 2 | ZP_06901810.1 | | | ZP_06901811 | | |  | |
| NAP08 | 3 | ZP_06890947.1 | | | ZP_06890946 ZP_06890948.1 | | |  | |
| NAP07 | 3 | ZP_06904810.1 | | | ZP_06904811 ZP_06904809.1 | | |  | |
| QCD-23m63 | 3 | ZP_05399847.1 | | | ZP_05399846 ZP_05399846.1 | | |  | |
| phiCDHM1 | 3 | This study | | |  | | |  | |
| *Staphylococcus aureus* subsp. aureus MRSA252 | - | YP_041486.1 | | | YP_041487.1 | YP_041488.1B | | YP_041489.1 | |

**Supporting Table S1. Strains and accession numbers for *agr* genes used in phylogenetic analysis.**
