# Supplementary material for: What Does the Talking?: Quorum Sensing Signalling Genes Discovered in a Bacteriophage Genome
Source: PLoS One. 2014 Jan 24;9(1):e85131. doi: 10.1371/journal.pone.0085131 (PMC3901668; doi:10.1371/journal.pone.0085131)
Supplement: Table S2 — Primers used for phage gene transcription PCR assays. (DOCX) [file pone.0085131.s005.docx]

**Supporting Table 2. Primers used for phage gene transcription PCR assays.**

| Target | Forward Primer | Reverse Primer | Product Size |
| --- | --- | --- | --- |
| Orf84 (+ve) | NTPRTF-5’ GAAGCACTTGGAAAACAAAGG ’3 | NTPRTR-5’ TCTTCGCAAGAAGCATCAAAA ’3 | 196 bp |
| Orf23 (+ve) | BPJRTF-5’ TGTTTGGAACGGTGGAGGTA ’3 | BPJRTR-5’ CCTTCTCCTTTCGGATTTGG ’3 | 119 bp |
| Orf76 (+ve) | CIRTF-5’ GGGAAGAAGAAGGCGAATACA ‘3 | CIRTR-5’ GCAAATATAGATGTTCTCCCT ’3 | 158 bp |
| *agrB* (Orf36, -ve) | ARGBRTF-5 CTTGCTCCAGTTTGCCACAT ‘3 | ARGBRTR-5’ TCACCCAAAATAAAGCTGAGG ‘3 | 171 bp |
| AgrC (Orf37, –ve) | WHKRTF-5’ TCAACAGTGTAAAAATTGGGA ‘3 | WHKRTR-5’ TGCTGTTAACAATAATGCCATA ’3 | 160 bp |

Abbreviations “+ve” = gene on the positive sense strand and “-ve” = gene on the negative sense strand.
