# Supplementary material for: What Does the Talking?: Quorum Sensing Signalling Genes Discovered in a Bacteriophage Genome
Source: PLoS One. 2014 Jan 24;9(1):e85131. doi: 10.1371/journal.pone.0085131 (PMC3901668; doi:10.1371/journal.pone.0085131)
Supplement: Table S3 — Strains and accessions of C. difficile isolates with phage agr genes. (DOCX) [file pone.0085131.s006.docx]

**Supporting Table 3. Strains and accessions of *C. difficile* isolates with phage *agr* genes.**

|  |  | aa sequence identity (%) | | | |
| --- | --- | --- | --- | --- | --- |
| Strain | Accession | AgrC |  | AgrD | AgrB |
| F200 | AVND01000019.1 | 100 |  | 100 | 100 |
| P24 | AVMA01000000 | 100 |  | 100 | 98 |
| CD45 | AVGY01000133.1 | 100 |  | 100 | 100 |
| DA00160 | AVJH01000078.1 | 99 |  | 98 | 98 |
| DA00193 | AVJO01000022.1 | 99 |  | 98 | 98 |
| DA00310 | AVKK01000614.1 | 99 |  | 99 | 98 |
| P2 | AVLM01000162.1 | 100 |  | 98 | 98 |
| CD42 | AVGV01000065.1 | 99 |  | 98 | 98 |
| CD159 | AVHV01000082.1 | 99 |  | 98 | 98 |
| CD165 | AVHX01000088.1 | 99 |  | 98 | 98 |
| DA00044 | AVIS01000043.1 | 99 |  | 98 | 98 |
| DA00154 | AVJG01000050.1 | 99 |  | 98 | 98 |
| DA00183 | AVJL01000040.1 | 99 |  | 98 | 98 |
| DA00261 | AVKE01000013.1 | 99 |  | 98 | 98 |
| P41 | AWZJ00000000.1 | 100 |  | 100 | 100 |
| DA00203 | AVJS01000001.1 | 99 |  | 98 | 98 |
| P19 | AVLW00000000.1 | 99 |  | 98 | 98 |
| P20 | AVLX0100000.1 | 99 |  | 98 | 98 |
| DA00275 | AVKG01000030.1 | 99 |  | 98 | 98 |
| F249 | AVKN01000101.1 | 99 |  | 98 | 98 |
| Y231 | AVLA01000014.1 | 99 |  | 98 | 98 |
| CD17 | AVGM01000142.1 | 100 | (partial) | 100 | 98 |
| P25 | AVMB01000008.1 | 100 | (partial) | 100 | 98 |
